# Supplementary material for: A methodology for the design of experiments in computational intelligence with multiple regression models
Source: PeerJ. 2016 Dec 1;4:e2721. doi: 10.7717/peerj.2721 (PMC5136129; doi:10.7717/peerj.2721)
Supplement: Data S1 — Datailed results from UC Irvine Machine Learning Repository (Housing, Machine CPU, Wine Quality, Automobile and Parkinson) and the 3 Use Cases (Protein Corona, Gajewicz Metal Oxides and Aquatic Toxicity) [file peerj-04-2721-s001.zip › 6_UseCases/2_GajewiczMetalOxides/SVMRFE.details.csv.repeatedcv.split2.pdf]

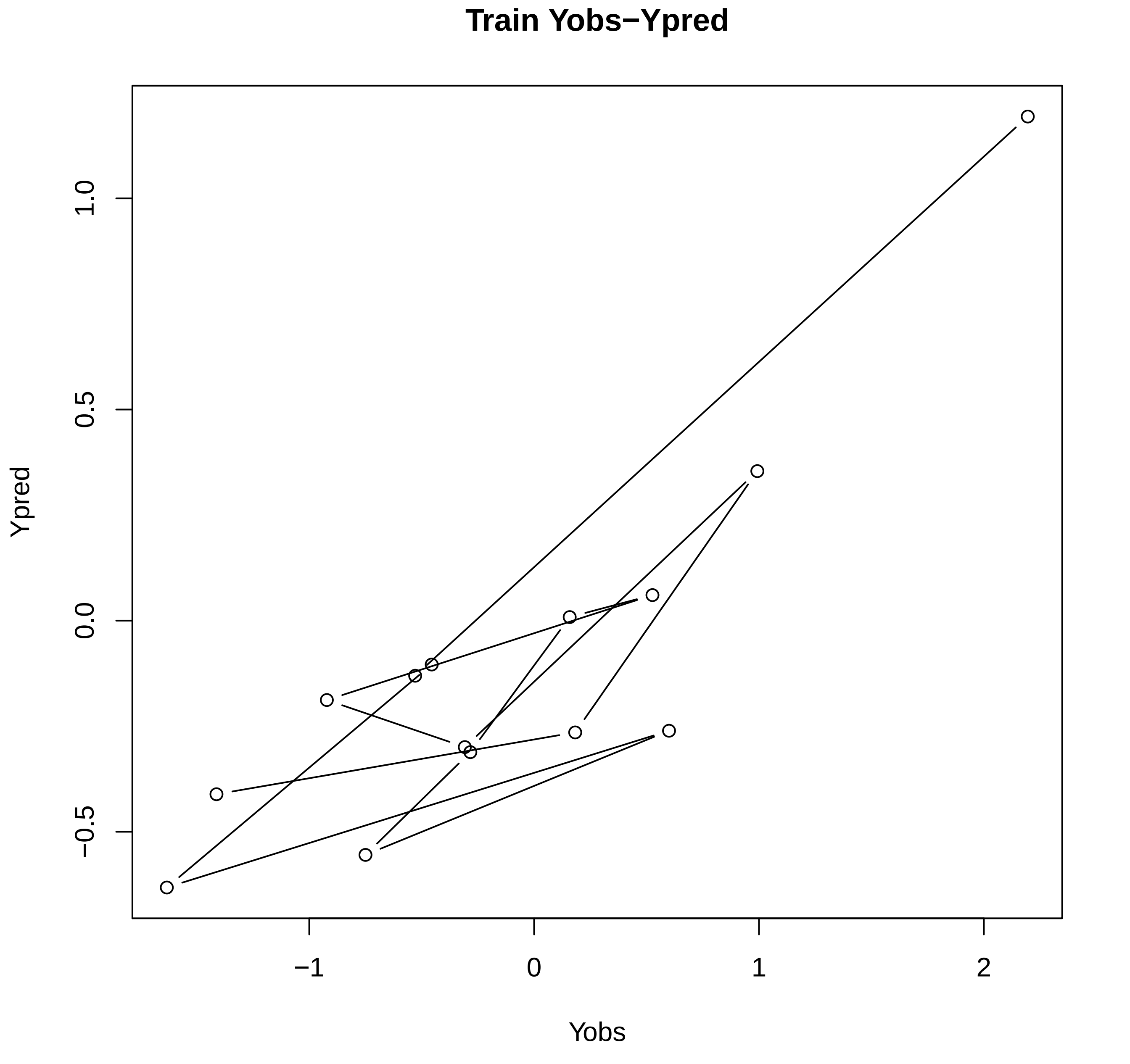
Train Yobs−Ypred
Ypred
1.0
0.5
0.0
−0.5
−1
0
1
2
Yobs

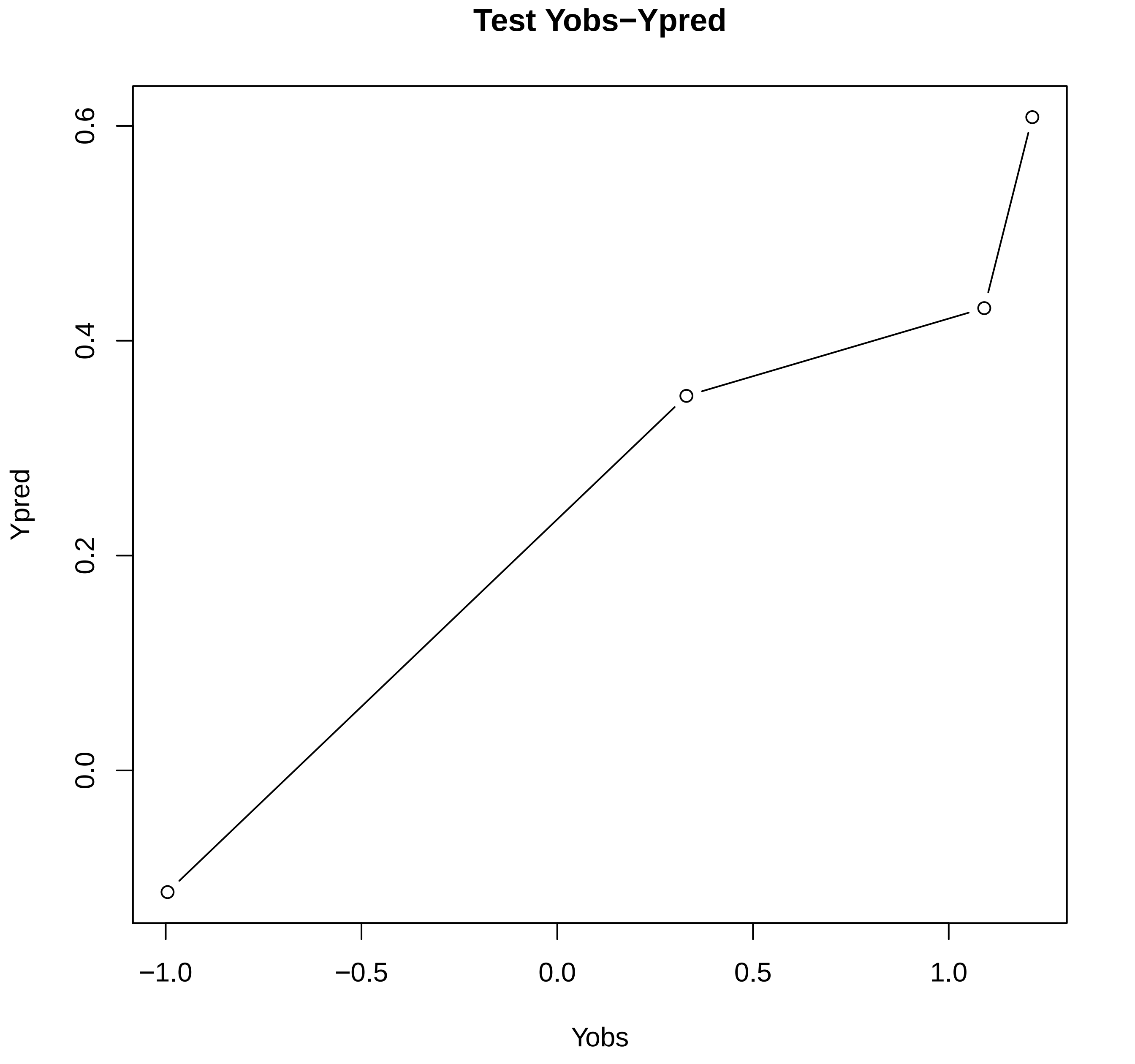
Test Yobs−Ypred
Ypred
0.0
0.2
0.4
0.6
Yobs
−1.0
−0.5
0.0
0.5
1.0

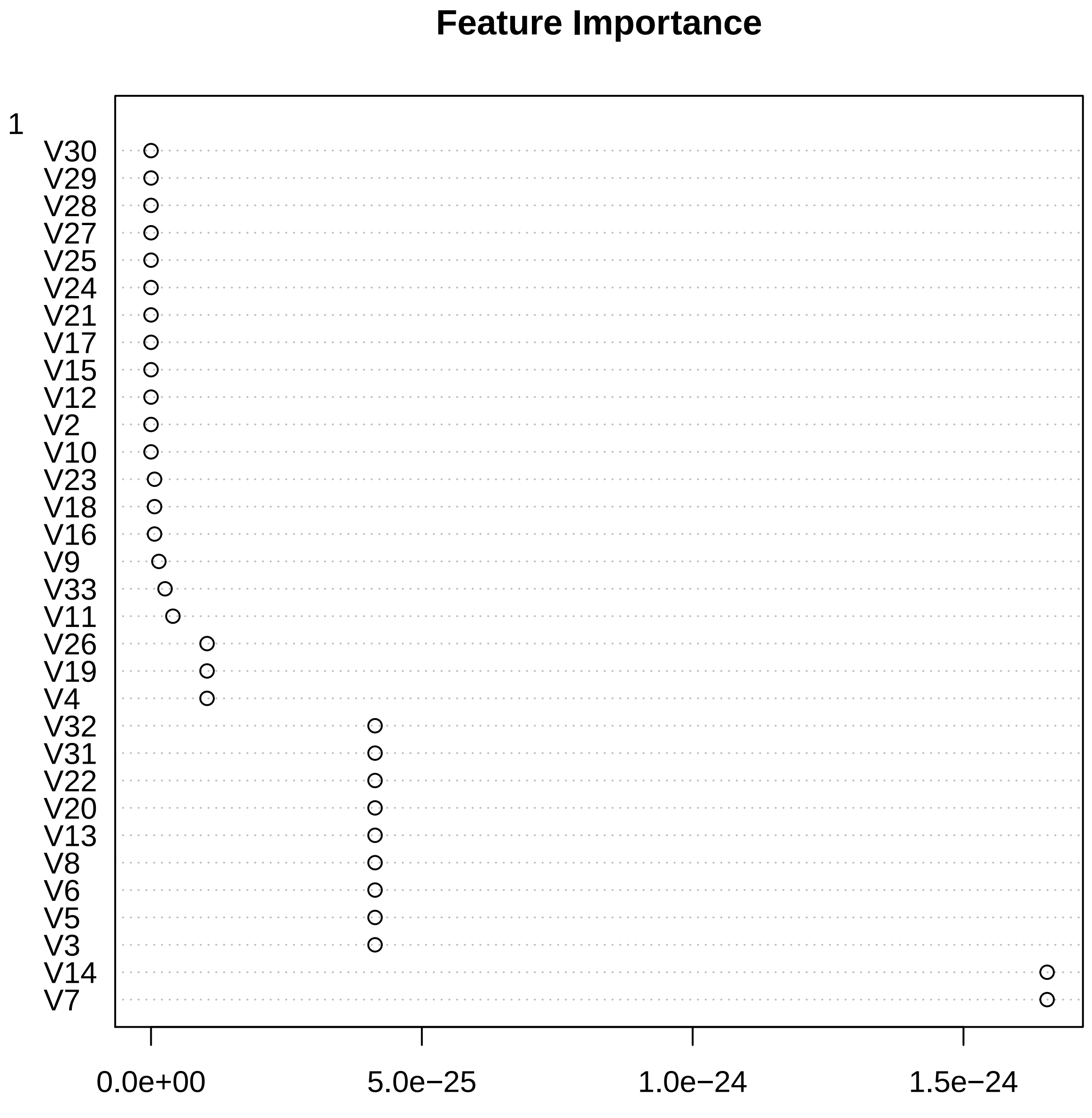

Feature Importance
1
V30
V29
V28
V27
V25
V24
V21
V17
V15
V12
V2
V10
V23
V18
V16
V9
V33
V11
V26
V19
V4
V32
V31
V22
V20
V13
V8
V6
V5
V3
V14
V7
0.0e+00
5.0e−25
1.0e−24
1.5e−24

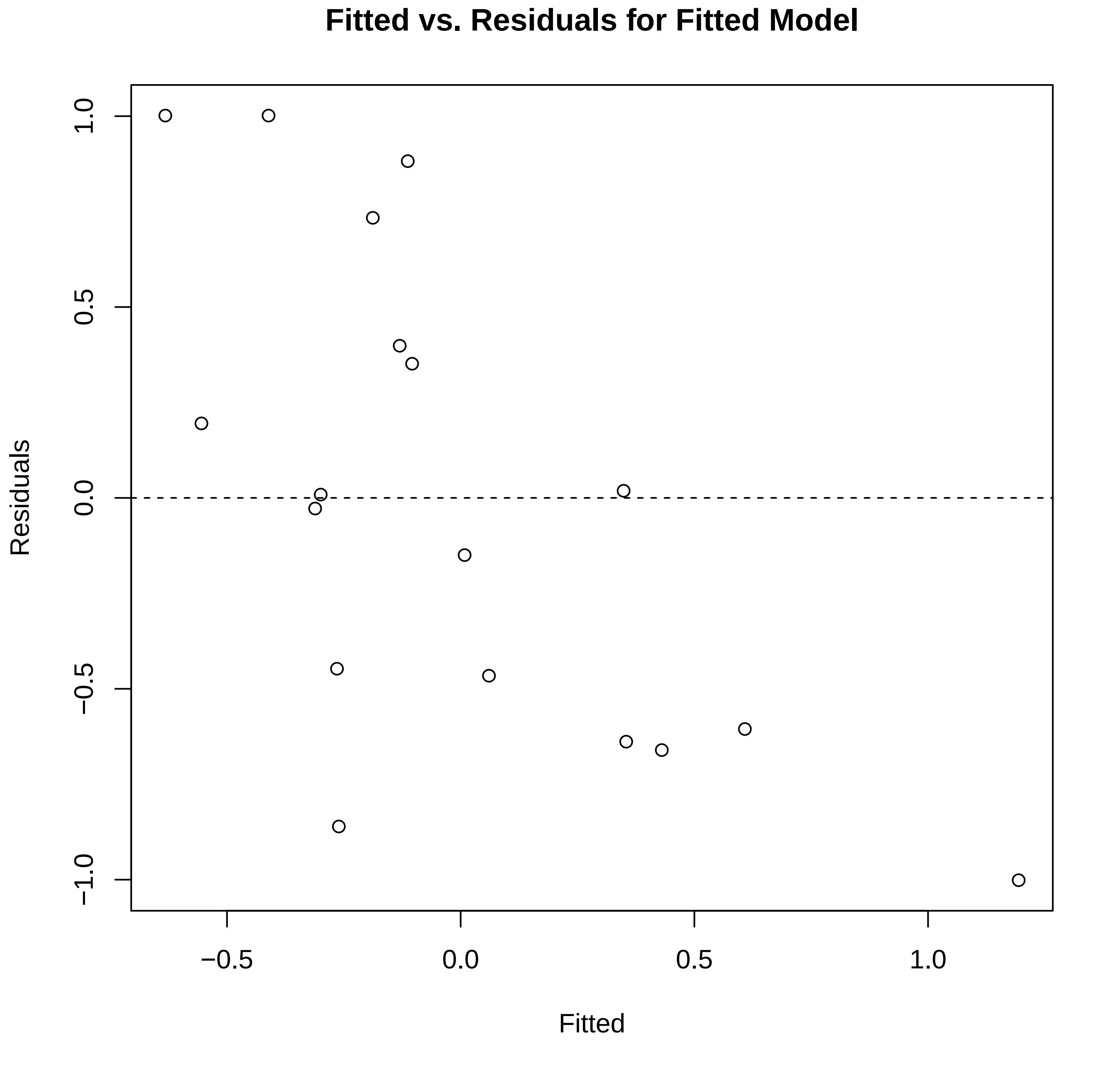
Fitted vs. Residuals for Fitted Model
Residuals
1.0
0.5
0.0
−0.5
−1.0
−0.5
0.0
0.5
1.0
Fitted
